# It Is Not Always Metastasis: A Case Report of a Primary Nervous System Anaplastic Large Cell Lymphoma and the Relevance of Brain Biopsy

**DOI:** 10.7759/cureus.98964

**Published:** 2025-12-11

**Authors:** Diego Salinas, Jisu Kim, Emilio A Sandoval, Barbara Saenz, Juan Eduardo Sebastian Aguirre Garza

**Affiliations:** 1 Internal Medicine, Hospital Universitario. "Dr. José Eleuterio González", Monterrey, MEX; 2 Dermatology, Hospital Zambrano Hellion, Tecnológico de Monterrey, San Pedro Garza García, MEX; 3 Pathology, Hospital Universitario. "Dr. José Eleuterio González", Monterrey, MEX

**Keywords:** anaplastic lymphoma, brain biopsy, brain tumor, chemotherapy, hematology, internal medicine, neurosurgery, oncology, pcnsl, seizures

## Abstract

Primary central nervous system lymphoma (PCNSL) is an uncommon, extranodal non-Hodgkin lymphoma confined to the brain, spinal cord, leptomeninges, or eyes. Most cases are diffuse large B-cell lymphomas; anaplastic large cell lymphoma (ALCL) in the central nervous system (CNS) is exceptional. We report an immunocompetent 63-year-old man with 3 months of intermittent bilateral pulsatile headaches who developed a focal seizure that secondarily generalized. He was started on levetiracetam for a first-time seizure and discharged. Outpatient magnetic resonance imaging (MRI) subsequently revealed a brain mass, prompting referral to our centre. On admission, he was hemodynamically stable; laboratory evaluation showed moderate lymphopenia and mild hyperphosphatemia. Brain MRI demonstrated an irregular extra-axial lesion in the left inferior frontal gyrus with punctate susceptibility foci and extensive vasogenic oedema involving adjacent frontal, insular, and temporal regions. The radiological impression favoured metastasis, and surgical resection was undertaken to obtain tissue and relieve mass effect. Histopathology and immunohistochemistry confirmed primary CNS ALCL, and a bone-marrow biopsy with CD30 immunostaining showed no systemic involvement. Postoperatively, new dysarthria and right hemiparesis developed. Two weeks later, high-dose methotrexate plus cytarabine was initiated, with neurological improvement. Twenty days after chemotherapy, he re-presented with fever, hypotension, and altered mental status. Computed tomography (CT) showed no new intracranial findings, but pancytopenia and cultures positive for carbapenem-resistant *Citrobacter freundii* were identified, and he died of septic shock. This case illustrates that CNS ALCL can radiographically mimic metastatic disease, underscores the necessity of tissue diagnosis, and highlights the competing imperatives of timely intensive chemotherapy and rigorous infection surveillance, particularly for multidrug-resistant organisms in older adults with PCNSL.

## Introduction

Primary central nervous system lymphoma (PCNSL) is a relatively rare disease, accounting for 2-6% of all primary brain malignancies and 1-2% of non-Hodgkin lymphomas. PCNSL is defined as lymphoma confined to the brain, spinal cord, leptomeninges, or eyes without systemic involvement. The vast majority of PCNSL cases are diffuse large B-cell lymphomas; T-cell central nervous system (CNS) lymphomas are exceptional, comprising less than 5% of cases, with reported frequencies ranging from 2% to 8.5% [1]. Primary CNS anaplastic large cell lymphoma (ALCL) is therefore sporadic, with a recent systematic review identifying only 39 reported cases [2]. Among these, 28 tumours were anaplastic lymphoma kinase (ALK) positive and 11 ALK negative, and age <40 years, ALK-positive tumour status, and methotrexate-based chemotherapy were associated with improved survival [2].

Clinical manifestations of PCNSL vary but often include headaches, focal neurological deficits, cognitive decline, and seizures. In reported primary CNS T-cell lymphomas, presenting symptoms include headache, aphasia, facial paralysis, sensory abnormalities, ataxia, leg weakness, and memory difficulties; the median age at presentation was 58.5 years (range 21-81 years) [1]. Diagnosis of PCNSL is challenging because imaging features are non-specific [3,4], and misdiagnosis and missed-diagnosis rates are high. Definitive diagnosis requires histopathological confirmation through stereotactic or open biopsy with immunohistochemical profiling. Standard therapy has not been firmly established, but high-dose methotrexate (HD-MTX)-based regimens are the cornerstone of treatment; CHOP (cyclophosphamide, doxorubicin, vincristine, and prednisone) chemotherapy has poor CNS penetration and is ineffective. Here, we describe a rare case of primary CNS ALK-positive ALCL in an immunocompetent 63-year-old man. This presentation lies outside the typical demographic profile and discusses diagnostic considerations, treatment strategy, and prognosis.

## Case presentation

A 63-year-old man presented with a 3-month history of intermittent bilateral pulsatile headaches. After one month of persistent symptoms, he developed a focal seizure that progressed to a generalized tonic-clonic seizure lasting approximately three minutes, followed by a 10-minute postictal phase. He was started on levetiracetam for a first-time seizure and discharged. Outpatient magnetic resonance imaging (MRI) subsequently revealed a brain mass, prompting referral to our institution.

He was admitted to the neurosurgery service while hemodynamically stable. Laboratory evaluation was notable for moderate lymphopenia (absolute lymphocyte count 0.5 × 10³/µL), a finding of potential significance in the context of a lymphoproliferative disorder and future immunosuppression, and mild hyperphosphatemia, as detailed in Tables 1-3.

**Table 1:** Complete blood count

Test	Result	Units	Reference Range
RBC	4.46	10^6/μL	4.04 – 6.13
Hemoglobin (HGB)	13.4	g/dL	12.20 – 18.10
Hematocrit (HCT)	41.4	%	37.7 – 53.7
Mean Corpuscular Volume (MCV)	92	fL	80 – 97
Mean Corpuscular Hemoglobin (MCH)	29.9	pg	27.0 – 31.2
Mean Corpuscular Hemoglobin Conc. (MCHC)	32.3	g/dL	29.9 – 34.2
Red Cell Distribution Width (RDW)	12.6	%	11.6 – 14.8
White Blood Cells (WBC)	4.87	10^3/μL	4.00 – 11.00
Neutrophils, absolute (NEU)	4.24	10^3/μL	2.00 – 6.90
Neutrophils, %	87	%	37.0 – 80.0
Lymphocytes, absolute (LYM)	0.5	10^3/μL	0.60 – 3.40
Lymphocytes, %	10.3	%	10.0 – 50.0
Monocytes, absolute	0.125	10^3/μL	0.000 – 0.900
Monocytes, %	2.57	%	0.00 – 12.00
Eosinophils, absolute	0	10^3/μL	0.000 – 0.700
Eosinophils, %	0	%	0.00 – 7.00
Basophils, absolute	0.011	10^3/μL	0.000 – 0.200
Basophils, %	0.21	%	0.00 – 2.50
Platelets (PLT)	268	10^3/μL	142 – 424
Mean Platelet Volume (MPV)	7.3	fL	7.4 – 10.4

**Table 2:** Blood clotting time

Test	Result	Units	Reference Range
Prothrombin Time (PT)	12.1	s	9.55 – 12.23
Partial Thromboplastin Time (aPTT)	27.8	s	28.0 – 35.6
International Normalized Ratio (INR)	1.11	—	0.8 – 1.2

**Table 3:** Blood chemistry

Test	Result	Units	Reference Range
A/G Ratio	1.23	—	≈1.0 – 2.2
Albumin	3.7	g/dL	3.2 – 5.5
Total Protein	6.7	g/dL	6.1 – 7.9
Globulin (Calculated)	3	g/dL	2.0 – 3.5
Uric Acid	3.3	mg/dL	4.8 – 8.7
Direct Bilirubin	0.1	mg/dL	0.0 – 0.2
Indirect Bilirubin	0.4	mg/dL	0.2 – 0.8
Total Bilirubin	0.5	mg/dL	0.2 – 1.0
Cholesterol	168	mg/dL	130 – 200
Creatinine	0.7	mg/dL	0.6 – 1.4
Phosphorus (Phosphate)	5	mg/dL	2.5 – 4.6
Glucose (Fasting)	103	mg/dL	60 – 100
Blood Urea Nitrogen (BUN)	11	mg/dL	7 – 20
Calcium	9.1	mg/dL	8.4 – 10.2
Triglycerides	80	mg/dL	35 – 150
Potassium	4.2	mmol/L	3.6 – 5.0
Sodium	138.8	mmol/L	135 – 145
Chloride	103.4	mmol/L	101 – 110
Serum Osmolality (Calculated)		mOsm/kg	280 – 295
Amylase	31	U/L	28 – 100
Alkaline Phosphatase (ALP)	89	U/L	38 – 126
Alanine Aminotransferase (ALT)	72	U/L	10 – 42
Aspartate Aminotransferase (AST)	26	U/L	10 – 42
Lactate Dehydrogenase (LDH)	143	U/L	91 – 180

**Figure 1:**
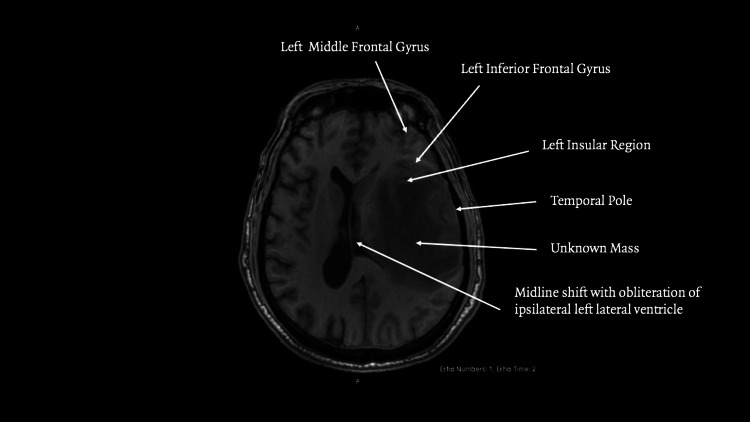
MRI axial view

**Figure 2:**
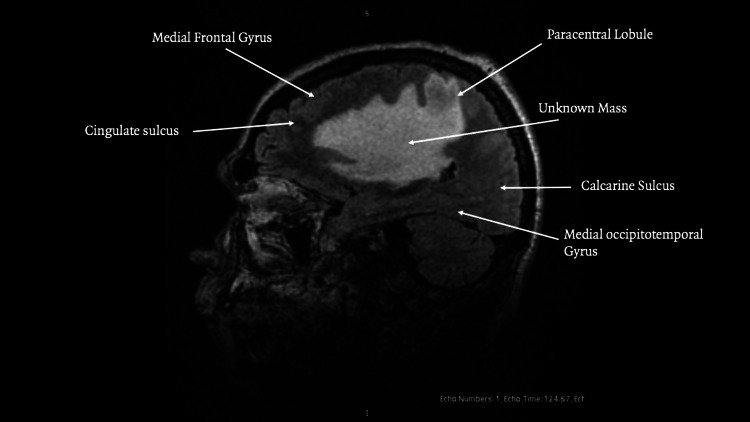
MRI sagittal view

**Figure 3:**
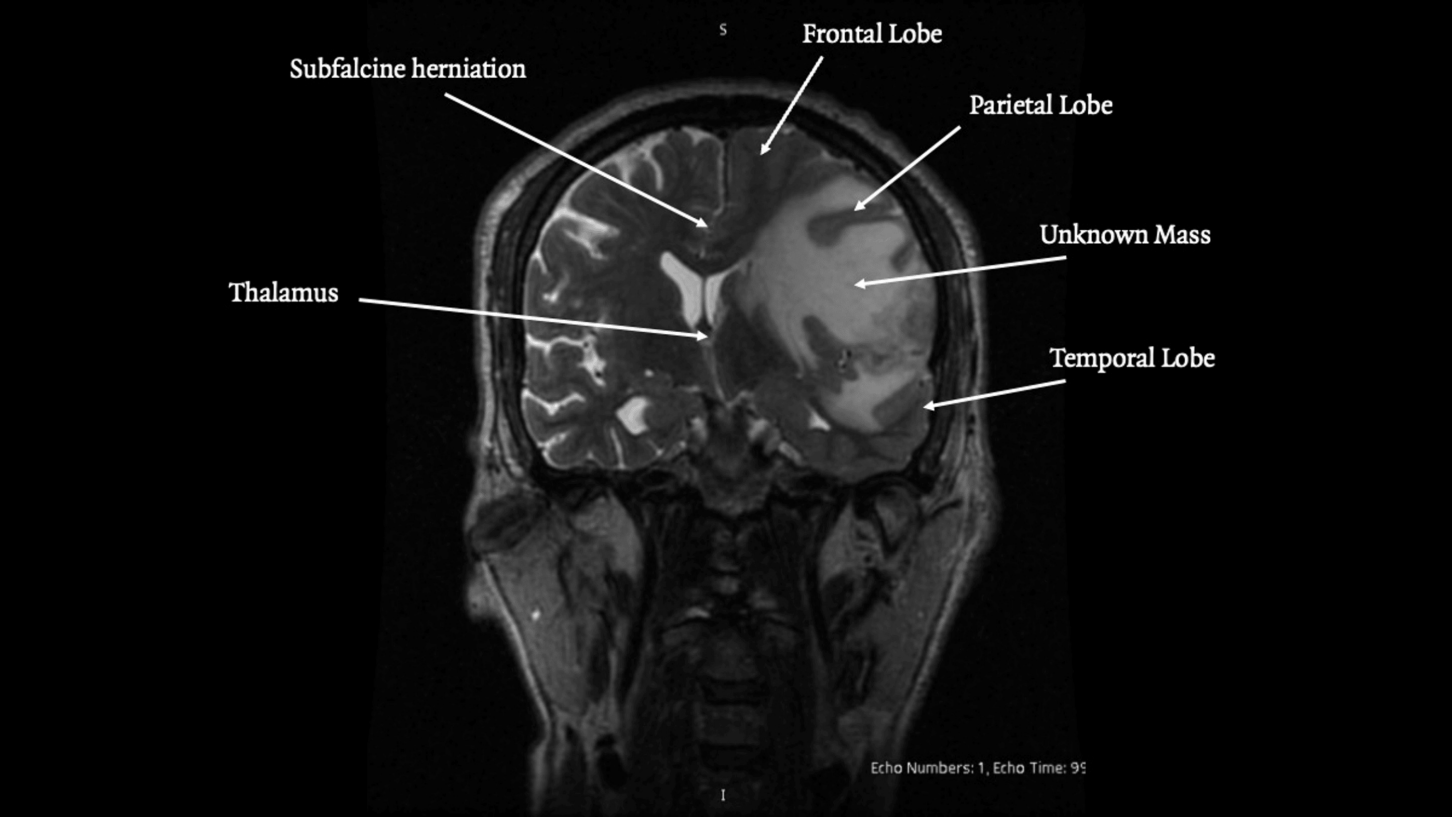
MRI coronal view

Imaging from a private clinic was not interpretable; repeat MRI at our institution demonstrated the findings seen in Figures 1-3.

An irregular extra‑axial lesion with well‑defined margins in the left inferior frontal gyrus. The lesion was hypointense on T1‑ and T2/fluid-attenuated inversion recovery (FLAIR)‑weighted sequences and contained punctate foci of magnetic susceptibility, suggesting hemosiderin. Extensive vasogenic edema involved the adjacent middle and inferior frontal gyri, insular region, and temporal pole, with effacement of the subarachnoid space in Figures 1-2. Post‑contrast images showed heterogeneous enhancement.

The lesion measured 25.9 × 8.9 × 23.6 millimeters with a semi‑automated segmentation volume of 5.7 cc. MR spectroscopy demonstrated decreased N-acetylaspartate with elevated choline, creatine, lipid, and lactate peaks, findings consistent with a high‑grade neoplastic process. Additional findings included left uncal and subfalcine herniation as seen in Figure 3, midline shift, and obliteration of the ipsilateral lateral ventricle. Whole‑body CT showed no extracranial disease.

**Figure 4:**
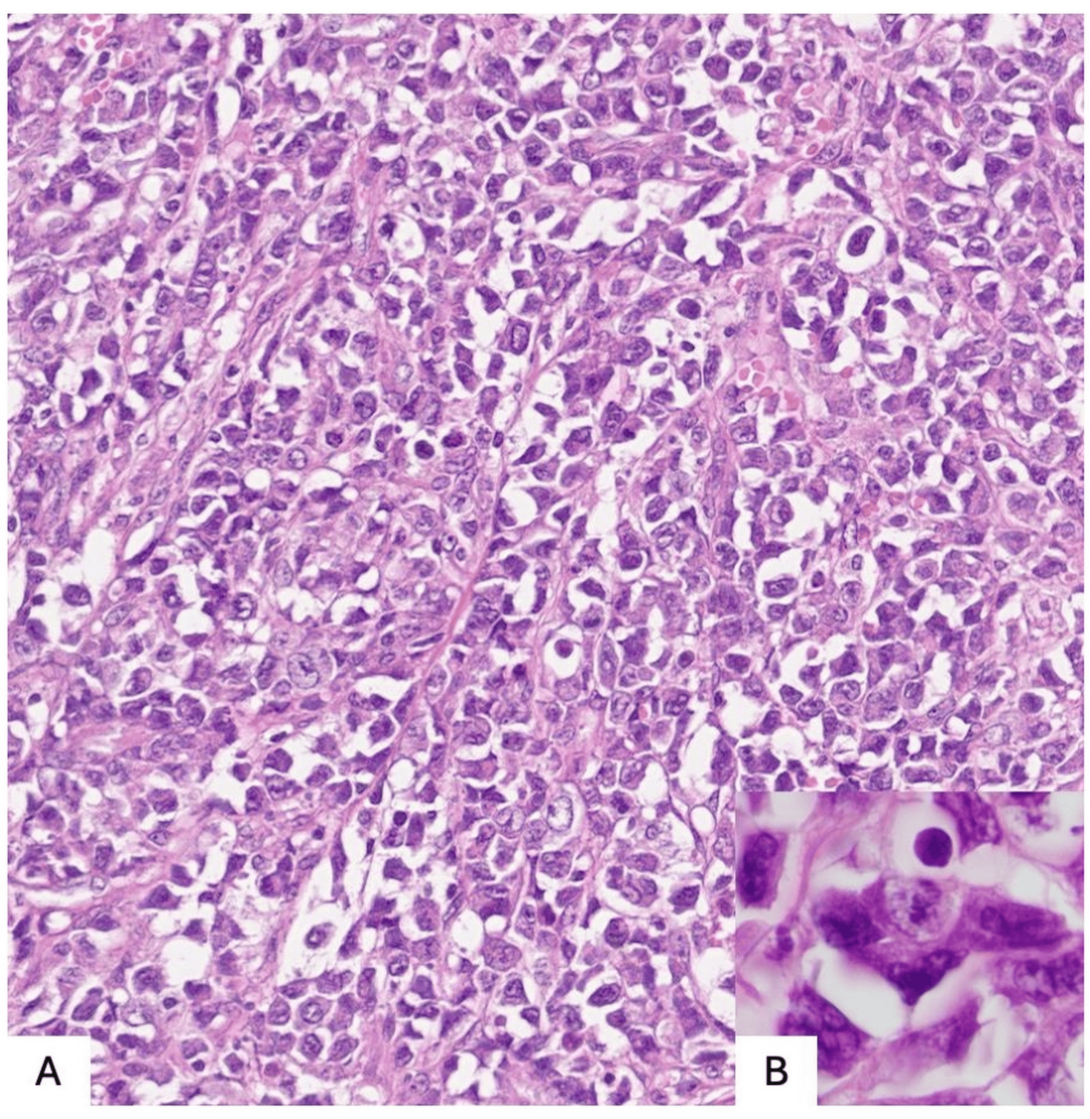
Neoplasm composed of large, discohesive cells with enlarged nuclei, prominent nucleoli, and scant cytoplasm, some of which exhibit anaplastic features A) Hematoxylin and Eosin staining in 200x B) Hematoxylin and Eosin staining in 1000x

**Figure 5:**
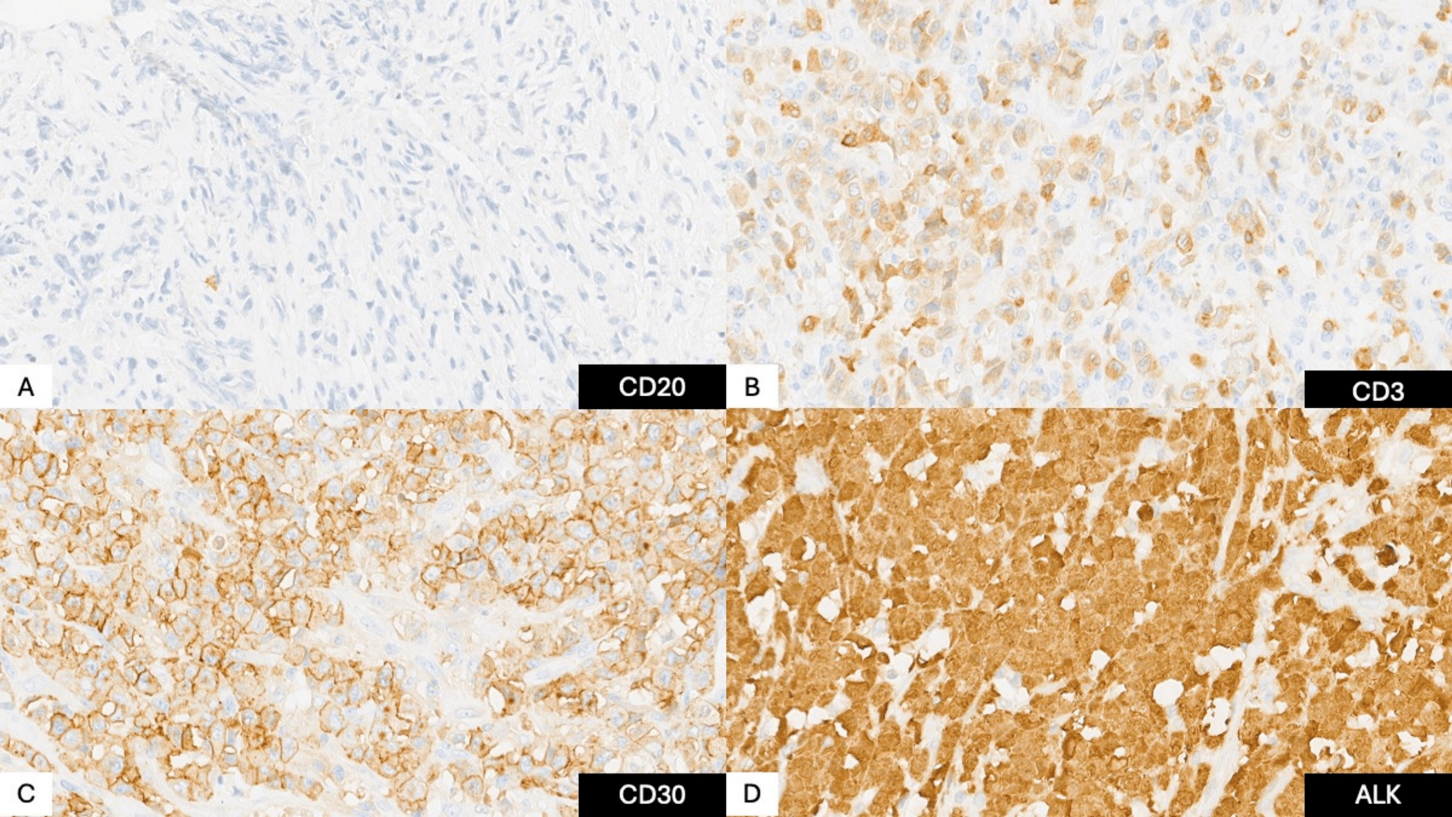
A) CD20 negative, B) CD3 cytoplasmic positive, C) CD30 membranous positive, D) ALK-positive block staining (nuclear and cytoplasmic) CD: cluster of differentiation; ALK: anaplastic lymphoma kinase

A surgical resection was performed to relieve mass effect and obtain tissue. Histopathology revealed a T‑cell anaplastic large cell lymphoma with diffuse large, pleomorphic lymphocytes strongly expressing cluster of differentiation (CD) 30; immunostaining for CD3, CD2, BCL‑6, and PU.1 was positive, and ALK was positive, as seen in Figures 4-5.

Bone‑marrow biopsy with CD30 immunohistochemistry showed no systemic infiltration, establishing the diagnosis of primary CNS ALCL.

Postoperatively, the patient developed new dysarthria and right hemiparesis, consistent with the expected sequelae of surgery in the left inferior frontal gyrus. He remained hospitalized for a week and was discharged with persistent deficits. Two weeks later, he was readmitted to begin chemotherapy. High-dose methotrexate in combination with cytarabine was administered over three days without complications. At discharge, dysarthria had resolved and hemiparesis had improved; he was able to ambulate with a walking frame.

Twenty days later, he presented to the emergency department with altered mental status, fever, tachycardia, and hypotension. Head CT showed no new intracranial findings. Laboratory testing revealed pancytopenia. Cultures were obtained, and empiric therapy for febrile neutropenia was initiated. Carbapenem‑resistant *Citrobacter freundii *harboring an NDM‑type metallo‑β‑lactamase was isolated from a rectal swab. Despite antimicrobial therapy and supportive care, he developed septic shock and died during this hospitalization.

## Discussion

The literature emphasises the rarity of T-cell PCNSL [5]. Univariate analyses have demonstrated that age <40 years, ALK positivity, and methotrexate-based chemotherapy are associated with improved survival [6,7]. Our patient’s presentation was unusual because, at 63 years old, he was significantly older than the median age of 17.5 years for ALK-positive CNS ALCL established in the literature [2], highlighting the disease’s potential to occur outside typical demographics.

Menon and colleagues reported that primary CNS T-cell lymphomas account for <5% of all CNS lymphomas, with reported frequencies varying between 2% and 8.5% across different countries. In their cohort of 18 cases, the median age was 58.5 years, and clinical presentations included headaches, aphasia, facial paralysis, sensory abnormalities, ataxia, leg weakness, and memory problems [5]. The largest compilation of primary CNS ALCL identified 39 cases, with 28 ALK-positive and 11 ALK-negative tumours; the median age was 17.5 years for ALK-positive tumours and 63 years for ALK-negative tumors, and the overall median age was 21 years [2].

Given the non-specific presentation and imaging features, PCNSL is frequently misdiagnosed or diagnosed late [8]. A recent narrative review highlighted that PCNSL lacks diagnostic specificity and that misdiagnosis and missed diagnosis rates are high [9]. Early biopsy and histopathological evaluation are therefore crucial.

Once the diagnosis is established, treatment generally follows PCNSL protocols. High-dose methotrexate is the backbone of therapy [10,11]. CHOP chemotherapy has poor CNS penetration and is ineffective. In reported cases of primary CNS ALCL, patients treated with high-dose methotrexate, often combined with cytarabine and/or whole-brain radiotherapy, experienced better outcomes than those who did not. Surgical resection is typically reserved for diagnostic purposes and to relieve mass effect; additional resection beyond biopsy does not confer a survival benefit. Nonetheless, in selected patients with solitary T-cell PCNSL, aggressive tumour debulking followed by systemic chemotherapy may be justified and has been associated with remission in some reports.

Primary CNS ALCL is exceptionally rare and poses a diagnostic challenge. Because the majority of PCNSL cases are diffuse large B-cell lymphomas, clinicians often first consider metastatic carcinoma or glioma when evaluating solitary brain lesions in older adults. In our case, the lesion’s extra-axial location and heterogeneous enhancement led to an initial suspicion of metastasis or meningioma. Such diagnostic pitfalls are not unique to lymphoma; they are a recurring issue across various CNS neoplasms. Previous literature has highlighted significant diagnostic challenges in hemangioblastoma, where, despite suggestive radiology, a definitive diagnosis remained elusive even post-autopsy, underscoring the universal limitations of imaging alone [12]. In both our case of ALCL and reported cases of other rare masses, no single imaging feature is pathognomonic; thus, tissue diagnosis is essential, as imaging cannot reliably distinguish these entities.

Our patient embodied a conflict of prognostic factors: his ALK-positive status and good performance status were favourable, but his advanced age placed him at higher risk for treatment-related toxicity. Ultimately, his death from a multidrug-resistant infection underscores the paramount importance of aggressive infection control and surveillance in older adults undergoing intensive chemotherapy, as this can outweigh even favorable tumor biology.

## Conclusions

This case highlights the importance of clinical vigilance when evaluating central nervous system masses. Even in older, immunocompetent patients, primary CNS lymphoma, including rare T‑cell subtypes, such as ALCL, must remain in the differential diagnosis. Because imaging cannot reliably distinguish PCNSL from more common intracranial lesions, stereotactic biopsy or surgical resection is essential for accurate diagnosis. Immunohistochemical assessment for ALK and CD30 not only confirms the diagnosis but also provides prognostic information. Although exceedingly rare, primary CNS ALCL can respond well to timely, high‑dose methotrexate-based therapy, and early initiation of treatment may improve outcomes. Heightened awareness of these atypical presentations can facilitate prompt diagnosis and management in this aggressive disease.
